# Atrial Fibrillation Is Not an Independent Determinant of Mortality Among Critically Ill Acute Ischemic Stroke Patients: A Propensity Score-Matched Analysis From the MIMIC-IV Database

**DOI:** 10.3389/fneur.2021.730244

**Published:** 2022-01-17

**Authors:** Chen-Shu Wu, Po-Huang Chen, Shu-Hao Chang, Cho-Hao Lee, Li-Yu Yang, Yen-Chung Chen, Hong-Jie Jhou

**Affiliations:** ^1^Department of Internal Medicine, National Defense Medical Center, Tri-Service General Hospital, Taipei, Taiwan; ^2^Department of Computer Science and Information Science, National Formosa University, Yunlin, Taiwan; ^3^Division of Hematology and Oncology Medicine, Department of Internal Medicine, National Defense Medical Center, Tri-Service General Hospital, Taipei, Taiwan; ^4^School of Medicine, Kaohsiung Medical University, Kaohsiung, Taiwan; ^5^Department of Neurology, Changhua Christian Hospital, Changhua, Taiwan; ^6^Department of Public Health, Chung Shan Medical University, Taichung, Taiwan

**Keywords:** ischemic stroke (IS), atrial fibrillation, intensive care unit (ICU), MIMIC-IV, propensity score matching (PSM)

## Abstract

**Background/Objective:**

This study was conducted to investigate the clinical characteristics and outcomes of patients with acute ischemic stroke and atrial fibrillation (AF) in intensive care units (ICUs).

**Methods:**

In the Medical Information Mart for Intensive Care IV database, 1,662 patients with acute ischemic stroke were identified from 2008 to 2019. Of the 1,662 patients, 653 had AF. The clinical characteristics and outcomes of patients with and without AF were compared using propensity score matching (PSM). Furthermore, univariate and multivariate Cox regression analyzes were performed.

**Results:**

Of the 1,662 patients, 39.2% had AF. The prevalence of AF in these patients increased in a stepwise manner with advanced age. Patients with AF were older and had higher Charlson Comorbidity Index, CHA2DS2-VASc Score, HAS-BLED score, and Acute Physiology Score III than those without AF. After PSM, 1,152 patients remained, comprising 576 matched pairs in both groups. In multivariate analysis, AF was not associated with higher ICU mortality [hazard ratio (HR), 0.95; 95% confidence interval (CI), 0.64–1.42] or in-hospital mortality (HR, 1.08; 95% CI, 0.79–1.47). In Kaplan–Meier analysis, no difference in ICU or in-hospital mortality was observed between patients with and without AF.

**Conclusions:**

AF could be associated with poor clinical characteristics and outcomes; however, it does not remain an independent short-term predictor of ICU and in-hospital mortality among patients with acute ischemic stroke after PSM with multivariate analysis.

## Introduction

Stroke is the second leading cause of death and the third leading cause of disability worldwide (1). Approximately 90% of stroke cases are ischemic stroke, resulting from arterial occlusion (2). The incidence of stroke increases with age, especially in low- and middle-income countries (3). A major cause of ischemic stroke is thrombosis and embolism from atherosclerotic plaque or from the heart. Compared with other mechanisms of ischemic stroke, patients with atrial fibrillation (AF), a specific risk factor for ischemic stroke, have worse clinical and imaging outcomes (4).

AF is the most prevalent chronic cardiac arrhythmia in the elderly with a reported prevalence of 1–2% in the general population (5, 6). Meanwhile, AF is a major cause of cardioembolic stroke, and patients with AF have a 4–5-fold higher risk of ischemic stroke than the general population (7). Presently, once the diagnosis of AF is made, oral anticoagulation treatment, such as apixaban, dabigatran, edoxaban, rivaroxaban, and warfarin, is recommended to reduce the risk of recurrent stroke, regardless of AF pattern according to the American Heart Association/American Stroke Association guidelines for preventing stroke in 2021 (2).

In a study, the long-term burden of AF resulted in complications, such as stroke, heart failure, and death (8). In another study by Saposnik et al., patients with ischemic stroke and AF had a higher risk of death and intracerebral hemorrhage than those without AF (9). However, some studies have shown that AF is not a predictor of mortality after adjustment using multivariable models (10). Older age and high stroke severity are factors explaining the association between AF and poorer outcomes after acute ischemic stroke (11). Additionally, the prevalence of AF increases by up to 25% for individuals aged more than 80 years (2, 12). A similar pattern was observed among critically ill patients in the intensive care unit (ICU) (13). However, whether AF in patients with stroke admitted to the ICU is associated with poor clinical outcomes remains unclear.

The inconsistency might be due to differences in settings or study designs. Besides, no studies have been conducted involving ICU patients, who had a higher prevalence of AF (14). We believe that using propensity score matching (PSM) presents a more authentic result of whether AF is an independent risk factor for mortality. Therefore, we conducted a retrospective study with PSM using detailed clinical data obtained from the Multiparameter Intelligent Monitoring in Intensive Care (MIMIC-IV) database to investigate the relationship between AF and the characteristics and clinical outcomes of patients with acute ischemic stroke who were admitted to the ICU.

## Methods

### Study Population and Data Source

We conducted a retrospective study based on the MIMIC-IV database (version 1.0) (15). This database is an updated version of MIMIC-III with preexisting Institutional Review Board approval (Massachusetts Institute of Technology, no. 0403000206; Beth Israel Deaconess Medical Center, 2001P001699) (16). Several improvements have been made, including simplifying the structure, adding new data elements, and improving the usability of previous data elements. Currently, the MIMIC-IV contains comprehensive and high-quality and de-identified data of patients admitted to the ICU or emergency department of the Beth Israel Deaconess Medical Center between 2008 and 2019. One author who has finished the Collaborative Institutional Training Initiative examination (certification number: 39050603 for author Jhou) can access the database and was responsible for data extraction.

### Study Population and Variable Extraction

The patients were identified in the MIMIC-IV database from 2008 to 2019. The inclusion criteria were as follows: adult patients (age, 18–89 years) with ischemic stroke, defined as ICD-9 codes of 433, 434, 436, 437.0, and 437.1 or ICD-10 codes of I63, I65, and I66. Patients with ischemic stroke who received acute reperfusion therapy, such as intravenous tissue plasminogen activator or endovascular mechanical thrombectomy were also enrolled in our analysis (17). The exclusion criteria were as follows: patients who were admitted to the ICU more than once, only data on the first ICU admission were recorded and used. The patients enrolled in this study were subsequently divided into the AF and non-AF groups.

The following baseline characteristics were identified: hypertension, hyperlipidemia, diabetes mellitus, coronary artery disease, congestive heart failure, peripheral vascular disease, chronic obstructive pulmonary disease, liver disease, peptic ulcer disease, chronic kidney disease, rheumatoid arthritis, dementia, malignancy, Acute Physiology Score III (APS III), CHA2DS2-VASc score, and HAS-BLED score (Supplementary Tables 1, 2) (18). The overall Charlson Comorbidity Index (CCI) encompassed 18 categories of medical conditions, which were identifiable in medical records (Supplementary Table 3) (19–21). Secondary prevention agents for ischemic stroke were also recorded and used, including antiplatelet agents (e.g., aspirin, clopidogrel, cilostazol, ticlopidine, ticagrelor, prasugrel, and dipyridamole) and anticoagulation agents (e.g., warfarin, dabigatran, rivaroxaban, apixaban, and edoxaban). Other clinical indicators included mean arterial pressure, heart rate, body temperature, saturation of peripheral oxygen, leukocyte count, hemoglobin, platelet, creatinine, blood urea nitrogen, sodium, potassium, and bilirubin within 24 h of ICU admission. When the aforementioned indicators had multiple results within 24 h, the worst value was recorded and used for the analysis.

### Outcome Measures

The major outcomes were ICU mortality and in-hospital mortality. The minor outcomes included the use of percutaneous endoscopic gastrostomy or jejunostomy tube placement and the complications of intracerebral hemorrhage.

### Statistical Analysis

Categorical variables were represented as number (percentage) and were compared using the chi-square test and Fisher's exact test. Continuous variables were described as means (standard deviation) and were compared using the independent samples *t*-tests or Wilcoxon rank-sum test (Mann–Whitney U-test). Missing values of each subjects were not defaulted to negative, and denominators were only reported cases.

Propensity scores were calculated involving the following preoperative variables: age, sex, CCI, APS III, CHA2DS2-VASc score, and HAS-BLED score. A logistic regression model was developed to estimate the patients' propensity scores for the AF group (22). PSM was performed using the Greedy 5-to-1 Digit-Matching algorithm between the AF and non-AF groups (23). In propensity-matched patients, univariate analyzes were conducted using the paired *t*-test or Wilcoxon signed-rank test for continuous variables and McNemar's test for categorical variables. Statistical testing was performed to evaluate the effectiveness of PSM. Both primary and secondary outcomes were compared based on the matched data. Sensitivity analysis was performed using the removal of new-onset AF (Supplementary Table 4).

For time-to-event outcomes, the major survival outcomes, the times that elapsed until the first event between the two groups were compared using the log-rank test, whereas the Kaplan–Meier method was used to estimate the absolute risk of each event for each group. Univariate and multivariate Cox hazards model analyzes were performed to identify the association between AF and major outcomes, and results were expressed as hazard ratio (HR) with 95% confidence interval (CI). The relationship between AF and minor outcomes was assessed using univariate and multivariate logistic regression analyzes, and results were expressed as odds ratio (OR) with 95% CI.

All comparisons were planned, the tests were 2-sided, and *p* < 0.05 were used to denote statistical significance. Statistical analyzes were performed using Statistical Package for the Social Sciences (version 25.0; IBM Corp., Armonk, NY, USA) and R (version 4.0.3; R Foundation for Statistical Computing, Vienna, Austria).

## Results

### Patient Characteristics

**Figure 1:**
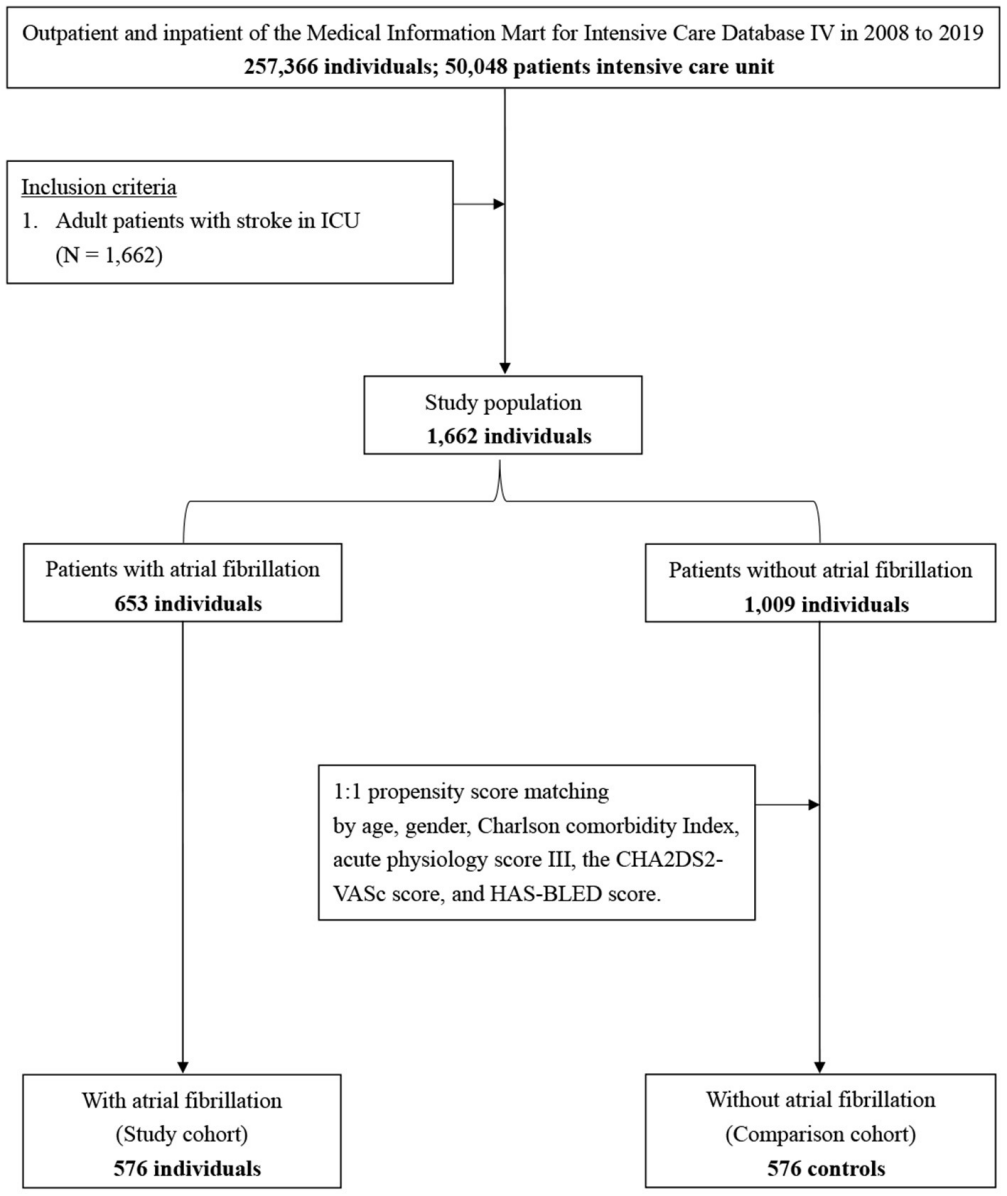
Flowchart of study sample selection from the Medical Information Mart for Intensive Care IV database.

Of the 257,366 medical records reviewed, 50,048 patients were admitted to the ICU. During the study period, we enrolled 1,662 patients with stroke, including 653 patients with AF and 1,009 patients without AF (Figure 1). There were 15 new-onset AF of those without history of AF (1.46%, 15/1,024). The basic demographic characteristics of the patients are shown in Table 1. The mean age of the AF and non-AF groups were 75.52 and 64.47 years, respectively. The prevalence of AF increased in a stepwise manner with advancing age, from 7.8% in those aged 50 years or younger to 63.5% in those older than 80 years (Figure 2). The AF group had higher CCI (7.66 vs. 6.35; *p* < 0.001), CHA2DS2-VASc score (5.49 vs. 4.60; *p* < 0.001), HAS-BLED score (4.02 vs. 3.53; *p* < 0.001), and APS III (44.27 vs. 35.77; *p* < 0.001) and had more comorbidities, including hyperlipidemia (25.4% vs. 19.8%; *p* = 0.007), congestive heart failure (30.9% vs. 11.6%; *p* < 0.001), chronic kidney disease (18.4% vs. 11.7%; *p* < 0.001), and dementia (5.7% vs. 3.4%; *p* = 0.024) (Table 1).

**Table 1:** Characteristics of the study patients.

**Characteristics**	**All patients**			**Propensity-matched pairs**		
	**AF group** **(***n*** = 653)**	**Non-AF group** **(***n*** = 1,009)**	***P*** **-value**	**AF group** **(***n*** = 576)**	**Non-AF group** **(***n*** = 576)**	***P*** **-value**
Age (years)	75.52 ± 10.59	64.47 ± 14.56	<0.001	74.30 ± 10.58	73.11 ± 10.21	0.052
**Gender**, ***n***			0.002			0.289
Male	312 (47.8%)	562 (55.7%)		283 (49.1%)	301 (52.3%)	
Female	341 (52.2%)	447 (44.3%)		293 (50.9%)	275 (47.7%)	
**Race**, ***n***			0.035			0.012
White	414 (63.4%)	621 (61.5%)		369 (64.1%)	361 (62.7%)	
Black	51 (7.8%)	120 (11.9%)		41 (7.1%)	70 (12.2%)	
Asian	21 (3.2%)	25 (2.5%)		20 (3.5%)	15 (2.6%)	
Other	167 (25.6%)	165 (24.1%)		146 (25.3%)	130 (22.5%)	
MAP (mmHg)	96.27 ± 18.58	94.93 ± 17.83	0.142	96.56 ± 18.53	93.28 ± 18.19	0.003
Temperature (°C)	36.69 ± 0.61	36.78 ± 0.54	0.001	36.69 ± 0.60	36.77 ± 0.57	0.035
Heart rate (beats/minute)	83.94 ± 20.22	78.51 ± 15.82	<0.001	83.66 ± 19.95	78.37 ± 16.24	<0.001
SpO_2_ (%)	97.23 ± 2.96	97.33 ± 2.79	0.501	97.27 ± 2.89	97.07 ± 3.14	0.250
**Comorbidities**, ***n***						
CCI	7.66 ± 2.30	6.35 ± 2.64	<0.001	7.54 ± 2.31	7.37 ± 2.45	0.230
Hypertension	484 (74.1%)	722 (71.6%)	0.253	423 (73.4%)	453 (78.6%)	0.038
Hyperlipidemia	166 (25.4%)	200 (19.8%)	0.007	145 (25.2%)	127 (22.0%)	0.212
**Diabetes mellitus**						
Without chronic complication	175 (26.8%)	272 (27.0%)	0.943	153 (26.6%)	167 (29.0%)	0.357
With chronic complication	44 (6.7%)	86 (8.5%)	0.186	38 (6.6%)	67 (11.6%)	0.003
Coronary artery disease	89 (13.6%)	110 (10.9%)	0.094	82 (14.2%)	77 (13.4%)	0.669
Congestive heart failure	202 (30.9%)	117 (11.6%)	<0.001	167 (29.0%)	75 (13.0%)	<0.001
PVD	68 (10.4%)	122 (12.1%)	0.294	59 (10.2%)	65 (11.3%)	0.568
COPD	120 (18.4%)	158 (15.7%)	0.147	109 (18.9%)	103 (17.9%)	0.648
**Liver disease**						
Mild	21 (3.2%)	24 (2.4%)	0.304	19 (3.3%)	8 (1.4%)	0.032
Moderate to severe	3 (0.5%)	5 (0.5%)	1.000^#^	3 (0.5%)	3 (0.5%)	1.000^#^
Peptic ulcer disease	10 (1.5%)	7 (0.7%)	0.097	7 (1.2%)	5 (0.9%)	0.562
Chronic kidney disease	120 (18.4%)	118 (11.7%)	<0.001	93 (16.1%)	96 (16.7%)	0.811
Rheumatoid disease	13 (2.0%)	27 (2.7%)	0.373	11 (1.9%)	20 (3.5%)	0.101
Dementia	37 (5.7%)	34 (3.4%)	0.024	28 (4.9%)	32 (5.6%)	0.596
Malignancy	41 (6.3%)	66 (6.5%)	0.831	37 (6.4%)	47 (8.2%)	0.257
**Laboratory parameters**						
WBC (10^9^/L)	10.14 ± 3.78	10.13 ± 4.59	0.938	10.15 ± 3.82	10.21 ± 4.77	0.821
Hgb (g/dL)	12.23 ± 2.23	12.38 ± 2.08	0.187	12.30 ± 2.20	12.13 ± 2.07	0.172
Platelet (10^9^/L)	222.96 ± 86.57	235.45 ± 91.12	0.006	222.13 ± 82.68	228.18 ± 82.30	0.225
Creatinine (mEq/L)	1.11 ± 0.96	1.03 ± 0.82	0.062	1.09 ± 0.98	1.10 ± 0.96	0.863
BUN (mg/dL)	21.07 ± 12.52	17.80 ± 11.57	<0.001	20.36 ± 12.19	20.42 ± 13.48	0.941
Sodium (mmol/L)	139.31 ± 4.25	139.34 ± 3.63	0.895	139.25 ± 4.10	139.35 ± 3.92	0.672
Potassium (mmol/L)	4.10 ± 0.60	4.03 ± 0.54	0.008	4.09 ± 0.58	4.06 ± 0.57	0.270
Bilirubin (mg/dL)	0.71 ± 0.51	0.60 ± 0.67	0.010	0.70 ± 0.50	0.61 ± 0.45	0.016
**Drugs**, ***n***						
Anti-platelet agents	469 (71.8%)	876 (86.8%)	<0.001	413 (71.7%)	499 (86.6%)	<0.001
**Anti-coagulation agents**						
Warfarin	168 (16.7%)	168 (16.7%)	<0.001	193 (33.5%)	68 (11.8%)	<0.001
NOAC	78 (11.9%)	17 (1.7%)	<0.001	75 (13.0%)	6 (1.0%)	<0.001
tPA or EVT	123 (27.3%)	140 (20.7%)	0.009	160 (27.8%)	116 (20.1%)	0.002
CHA2DS2-VASc score	5.49 ± 1.37	4.60 ± 1.51	<0.001	5.38 ± 1.36	5.27 ± 1.38	0.169
HAS-BLED score	4.02 ± 0.93	3.53 ± 0.92	<0.001	3.95 ± 0.93	3.93 ± 0.80	0.634
APS III	44.27 ± 20.11	35.77 ± 17.34	<0.001	42.40 ± 19.24	40.79 ± 18.69	0.148
ICU mortality, *n*	75 (11.5%)	65 (6.4%)	<0.001	59 (10.2%)	50 (8.7%)	0.365
ICU length of stay, day	4.90 ± 6.61	3.91 ± 4.55	<0.001	4.82 ± 6.58	3.87 ± 4.74	0.005
In-hospital mortality, *n*	125 (19.1%)	102 (10.1%)	<0.001	95 (16.5%)	85 (14.8%)	0.417
Hospital length of stay, day	9.26 ± 10.06	7.39 ± 8.43	<0.001	9.15 ± 10.08	7.36 ± 8.79	0.001
Intracranial hemorrhage, *n*	110 (16.8%)	86 (8.5%)	<0.001	90 (15.6%)	53 (9.2%)	0.001
PEG/PEJ tube placement, *n*	113 (17.3%)	95 (9.4%)	0.001	99 (17.2%)	57 (9.9%)	<0.001

*Propensity score matching by age, sex, Charlson comorbidity Index, acute physiology score III, the CHA2DS2-VASc score, and HAS-BLED score*.

*APS III, acute physiology score III; BPM, beats per minute; BUN, blood urea nitrogen; CCI, Charlson comorbidity Index; COPD, chronic obstructive pulmonary disease; Hgb, hemoglobin; MAP: mean arterial pressure; NOAC, novel oral anticoagulant; PVD, Peripheral vascular disease; SpO_2_, saturation of peripheral oxygen; PEG, percutaneous endoscopic gastrostomy; PEJ, percutaneous endoscopic jejunostomy; WBC, white blood cell*.

# *Testing by Fisher exact test or Wilcoxon Test, respectively*.

**Figure 2:**
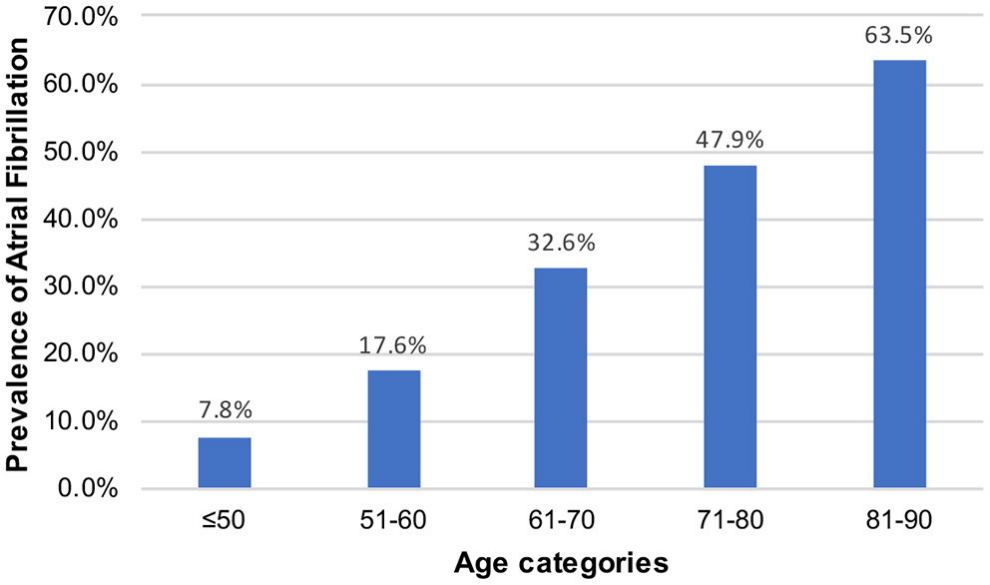
Prevalence of atrial fibrillation.

### Post-PSM Characteristics

**Figure 3:**
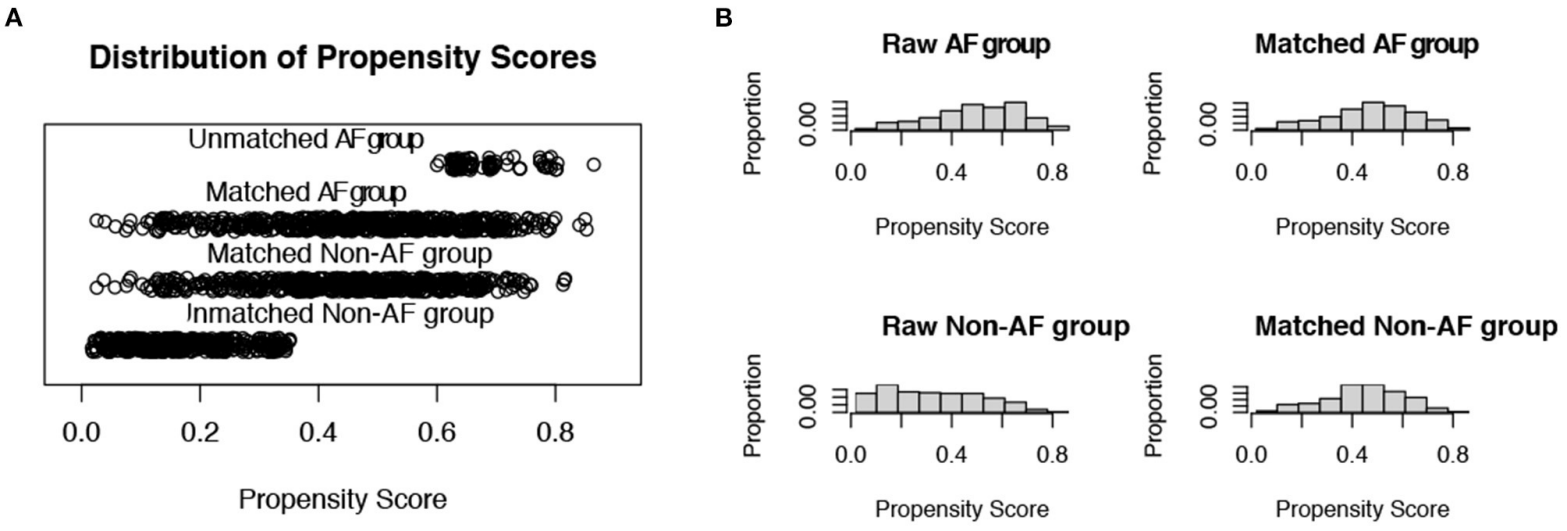
Distribution of propensity scores. **(A)** Jittered plot presenting matched and unmatched subjects, and their distribution of propensity score values; **(B)** Histograms demonstrating the density of propensity score distribution in the atrial fibrillation group and the non-atrial fibrillation group before and after matching. AF, atrial fibrillation.

Propensity scores were calculated involving the following covariates: age, sex, CCI, APS III, CHA2DS2-VASc score, and HAS-BLED score. After 1:1 PSM, 576 pairs in each arm remained. The cohorts were well-balanced based on six covariates between the AF and non-AF groups (Figure 3).

### Kaplan–Meier Survival Curve of Primary Outcomes Between the AF and Non-AF Groups

**Figure 4:**
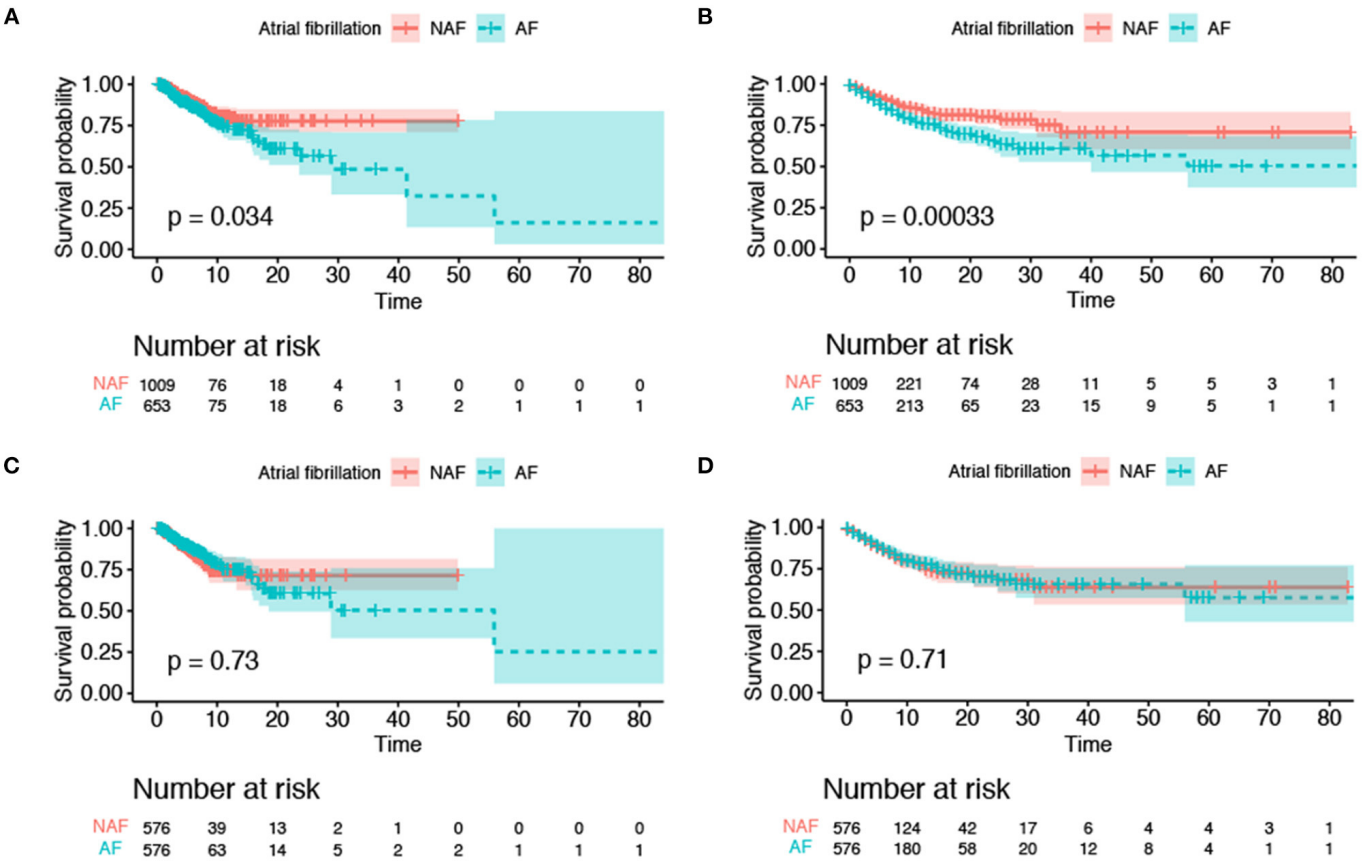
Kaplan–Meier survival curves for survival to intensive care unit discharge and hospital discharge. **(A)** ICU mortality before propensity score matching; **(B)** in-hospital mortality before propensity score matching; **(C)** ICU mortality after propensity score matching; **(D)** in-hospital mortality after propensity score matching. The colored area was the standard deviation. ICU, intensive care unit.

The AF group had a higher risk of ICU mortality (11.5% vs. 6.4%; *p* < 0.001) and in-hospital mortality (19.1% vs. 10.1%; *p* < 0.001) than the non-AF group (Table 1). The Kaplan–Meier curves for ICU discharge and survival between the AF and non-AF groups are shown in Figures 4A,B, and both of these curves were significantly different. However, no difference in hospital discharge and survival was observed between patients with and without AF (Figures 4C,D). Survival was followed until hospital discharge, and the longest length of hospital stay was 88 days.

### Outcome Measurement

In the cohort before PSM, univariate Cox regression analysis revealed significant difference in ICU mortality (crude HR, 1.43; 95% CI, 1.03–2.00) and in-hospital mortality (crude HR, 1.61; 95% CI, 1.24–2.09); furthermore, patients with AF had a higher risk of intracerebral hemorrhage (crude OR, 2.17; 95% CI, 1.61–2.94) and percutaneous endoscopic gastrostomy (PEG) or percutaneous endoscopic jejunostomy (PEJ) placement before PSM (crude OR, 2.01; 95% CI, 1.50–2.70).

After PSM, the results of univariate Cox regression analysis showed that AF was not associated with higher ICU mortality (HR, 0.94; 95% CI, 0.64–1.37) and in-hospital mortality (crude HR, 0.95; 95% CI, 0.71–1.27). In minor outcomes, patients with AF remained a higher risk of intracranial hemorrhage and PEG/PEJ tube placement with crude ORs of 1.83 (95% CI, 1.27–2.62) and 1.89 (95% CI, 1.33–2.68), respectively.

After multivariate analysis, after adjusting for race, hypertension, congestive heart failure, liver disease, diabetes, anti-platelet agents, anti-coagulation agents, intravenous tissue plasminogen activator or endovascular mechanical thrombectomy, no differences in ICU mortality (adjusted HR, 0.95; 95% CI, 0.64–1.42), in-hospital mortality (adjusted HR, 1.08; 95% CI, 0.79–1.47). However, the risk of intracerebral hemorrhage (adjusted OR, 1.96; 95% CI, 1.31–2.92), and PEG or PEJ tube placement before PSM (adjusted OR, 1.76; 95% CI, 1.19–2.60) remained higher among patients with AF than those without AF. The results were shown in Table 2. In the sensitivity analyses, we noted the similar findings before and after PSM cohort, as well as adjusted PSM cohort (Supplementary Table 4).

**Table 2:** Association between outcomes and atrial fibrillation among patients with ischemic stroke.

	**With atrial fibrillation vs. Without atrial fibrillation (Reference)**					
**Outcomes**	**Before PSM—Univariate**		**After PSM—Univariate**		**After PSM—Multivariate**	
	**Crude HR (95% CI)**	***P*** **-value**	**Crude HR (95% CI)**	***P-*** **value**	**Adjusted HR (95% CI)** ^#^	***P*** **-value**
ICU Mortality	1.43 (1.03–2.00)	0.035	0.94 (0.64–1.37)	0.733	0.95 (0.64–1.42)	0.806
In-hospital Mortality	1.61 (1.24–2.09)	<0.001	0.95 (0.71–1.27)	0.713	1.08 (0.79–1.47)	0.630

*Propensity score matching by age, sex, Charlson comorbidity Index, acute physiology score III, the CHA2DS2-VASc score, and HAS-BLED score*.

*HR, hazard ratio; OR, odds ratio; PEG, percutaneous endoscopic gastrostomy; PEJ, percutaneous endoscopic jejunostomy*.

# *All results of OR were adjusted by race, coronary artery disease, congestive heart failure, peripheral vascular disease, dementia, chronic obstructive pulmonary disease, rheumatic arthritis, peptic ulcer disease, liver disease, diabetes, renal disease, paraplegia, malignancy, metastatic solid tumor, anti-platelet agents, anti-coagulation agents*.

## Discussion

In this study, we adapted the latest MIMIC-IV database, including medical records of ICU-admitted patients from 2008 to 2019, and used the PSM method for preprocessing data for causal inference. We demonstrated that AF might be a risk factor for ICU or in-hospital mortality among ICU patients with acute ischemic stroke, but it is incoherent through multivariate analysis. Furthermore, secondary outcome analysis demonstrated ICU patients with AF had higher risk of ICH or PEG/PEJ tube placement than those without AF.

Preexisting or new-onset AF is more common among critically ill patients in the ICU (13). The prevalence of AF has been underestimated due to difficulty detecting paroxysmal AF. The mean age of the patients in this study was 68.81 years, which is identical to that (63.9–76.2 years) in a previous study (24–27). The prevalence of AF increases markedly with increasing age (28). In a study from Swedish, the prevalence of AF in patients with ischemic stroke increased with age from 8.6% at <60 years to ≥50% at >90 years (29). This study showed a similar trend, indicating an adequate population representativeness. The prevalence increased in a stepwise manner with advancing age, from 7.8% in those aged 50 years or younger to 63.5% in those older than 80 years. We found an overall AF frequency of 39.3% among patients with ischemic stroke, which is higher than the range of 9.1–31% reported in other studies (10, 11, 30–33), and this may be due to prolonged electrocardiography monitoring and the development of new-onset AF under critical illness (34).

In the past, the prospective follow-up of the Framingham Study cohort failed to observe an association between AF at baseline and the subsequent risk of fatal stroke in 1983 (35). In the previous study conducted by Oxfordshire Community Stroke Project (36), which demonstrated that AF was associated with an increased risk of death within acute phase of stroke in 30 days outcome, however, those excess risk might be explained by the older age or more comorbidities, such as diabetes, in patients with AF. Otherwise, in the long-term prognosis, risk of death from all causes did not show any difference between AF and sinus rhythm group in the multiple regression analyses. Those results would be consistent with our study. Furthermore, investigators of the FINMONICA Stroke Register had analyzed stroke with AF from 1982 to 1992 (37); after simply adjusting with multivariate regression for age, sex, and comorbidities, they have concluded that AF is associated with higher mortality in patients with stroke. This study showed an accordant trend before preprocessing data using the PSM method.

However, the mortality of patients with stroke is influenced by several factors. The older the patients, the higher the prevalence of AF (38, 39). This study showed an identical tendency that the patients in the AF group had more comorbidities and were older than those in the non-AF group. Older patients with stroke have more disabling strokes and higher mortality rate (40, 41). Additionally, elderly people with AF are less likely to receive oral anticoagulation (OAC) therapy, which could substantially reduce the risk of death and severe disability after ischemic stroke in patients with AF (11, 42). This is mainly due to concerns on a higher risk of OAC-associated hemorrhage in the elderly population (43). Therefore, increased age has always been a factor for mortality after stroke. Moreover, differences in mortality after stroke between sexes might be another important issue, and women are reported to have greater mortality after stroke than men (44). Both the higher CHA2DS2-VASc and HAS-BLED scores were associated with and became significant predictors of mortality in patients with AF (45). The CCI score was independently associated with mortality after a cerebrovascular event, for example, ischemic stroke (46).

In this study, before preprocessing data, severe heterogeneity was observed between patients with and without AF; thus, we adapted the PSM method to eliminate the potential influence of confounding factors. Propensity scores have been proposed as a method for equating groups at baseline, especially in studies that do not use randomization. They can be thought of as a balancing score that, like random assignment, attempts to balance the distribution of these measured covariates between two groups (47). Hence, we adapted the PSM method in age, sex, and scoring systems, including APS III, CHA2DS2–VASc, HAS-BLED, and CCI scores, to obtain a balanced distribution of generalized conditions and to eliminate heterogeneity between the two groups. Thus, we adjusted the detailed covariates between the compared groups using multivariate regression models to maximize the bias-reducing mechanism. After PSM and the development of multivariate regression models, no difference in mortality rate was found, indicating that age, sex, and comorbidities caused the misleading association between AF and a higher mortality rate.

Recently, Gattringer et al. have developed a simple score to estimate the early mortality of patients with ischemic stroke and found that AF, which was clearly associated with mortality in univariate analysis, did not remain an independent predictor of stroke-related mortality after multivariate analysis (48). Therefore, AF was no longer listed as one of the scoring items in predicting early mortality in patients with acute ischemic stroke.

Cerebral infarction weakens the blood–brain barrier, increasing the risk of spontaneous hemorrhage after acute ischemic stroke (49). As patients with the history of ischemic stroke use anticoagulants for secondary prevention, it reduces the risk of recurrent embolism (50). Several meta-analyses also showed that reperfusion therapy might have proportional benefits; however, the risk of fatal intracranial hemorrhage and reperfusion injury remain high during the first few days after treatment (51). In this study, patients with stroke and AF had a risk of intracerebral hemorrhage, even after PSM with adjustment of comorbidities, anticoagulants, and reperfusion therapy. Regarding disabilities after stroke, Alkhouli et al. have shown that patients with ischemic stroke with AF were more likely to undergo gastric tube placement than those without AF (52). Similar findings were observed in this study; moreover, in the matched groups with adjustment model, patients with ischemic stroke with AF also had a higher risk of PEG/PEJ tube placement.

### Limitations and Strengths

The main strength of this study is its large-scale, diverse population study design using real-world data. However, the results should be interpreted in the context of the following limitations. First, the study design was retrospective, and the diagnosis of ischemic stroke relied solely on administrative diagnosis codes. We could not confirm the diagnostic accuracy by evaluating the patients directly. Therefore, misclassifications could lead to false associations. Second, although we adjusted as much bias as we could by using PSM and multivariable analysis to balance the baseline differences between the groups and eliminate residual confounding effects, biases related to unmeasured confounders remain a potential issue in this study. Third, given the nature of the MIMIC database, we lacked some potential factors on the National Institute of Health Stroke Scale, the subtypes of ischemic stroke (TOAST classification), onset time of stroke, the timing of AF diagnosis, the categorization of AF type, cardiac function parameters, and the mortality cause. No long-term follow-up events remained; therefore, the 3-month modified Rankin Scale score cannot be measured. Fourth, this was a single-institution study, and selection bias of patients might exit. Because of a narrow window time after symptom onset of ischemic stroke for reperfusion therapy and the evolution of stroke severity, some patients might not be referred from other hospitals. These high-risk patients might not have been included in our cohort. Finally, although novel OAC (NOAC) and warfarin were used for the secondary prevention of stroke in patients with AF, we could not obtain the actual and detailed information or reasons as to why physicians choose to prescribe warfarin or NOACs based on the administrative database, and thus, we could not add these potential factors into the propensity score models or adjust for these factors in our regression models. Furthermore, we enrolled the patients with AF and diagnosed as ischemic stroke in the intensive care unit, and the phase of the stroke diagnosis was not clear. Whether the patient was under an acute stroke phase might have an influence on the dissociation of poor clinical outcome and in-hospital mortality, which would be an another limitation to our study. Because this study focused on the ICU setting, further studies are warranted to examine the external generalizability of our results.

## Conclusion

This retrospective study showed that patients with acute ischemic stroke with AF have poor clinical characteristics and prognosis compared with those without AF. However, AF might not be an independent risk factor for patients with acute ischemic stroke after adjusting for stroke-related comorbidities.

## Data Availability Statement

The original contributions presented in the study are included in the article/Supplementary Material, further inquiries can be directed to the corresponding author/s.

## Ethics Statement

The Multiparameter Intelligent Monitoring in Intensive Care-IV database was approved by the Institutional Review Board (IRB) of the Beth Israel Deaconess Medical Center (IRB Protocol #2001P001699) and was granted a waiver of informed consent. Written informed consent for participation was not required for this study in accordance with the national legislation and the institutional requirements.

## Author Contributions

Y-CC and H-JJ conceptualized the research goals, planned the analyses, and guided the literature review. S-HC extracted the data from the MIMIC-IV database. C-HL, L-YY, and H-JJ participated in processing the data and doing the statistical analysis. C-SW and P-HC wrote the first draft of the paper. All authors have read and approved the final manuscript.

## Funding

Financial support of this study was from Tri-Service General Hospital/National Defense Medical Centre (No. TSGH-B-111157).

## Conflict of Interest

The authors declare that the research was conducted in the absence of any commercial or financial relationships that could be construed as a potential conflict of interest.

## Publisher's Note

All claims expressed in this article are solely those of the authors and do not necessarily represent those of their affiliated organizations, or those of the publisher, the editors and the reviewers. Any product that may be evaluated in this article, or claim that may be made by its manufacturer, is not guaranteed or endorsed by the publisher.
