# Supplementary figures and images for: Atrial Fibrillation Is Not an Independent Determinant of Mortality Among Critically Ill Acute Ischemic Stroke Patients: A Propensity Score-Matched Analysis From the MIMIC-IV Database
Source: Front Neurol. 2022 Jan 17;12:730244. doi: 10.3389/fneur.2021.730244 (PMC8801535; doi:10.3389/fneur.2021.730244)

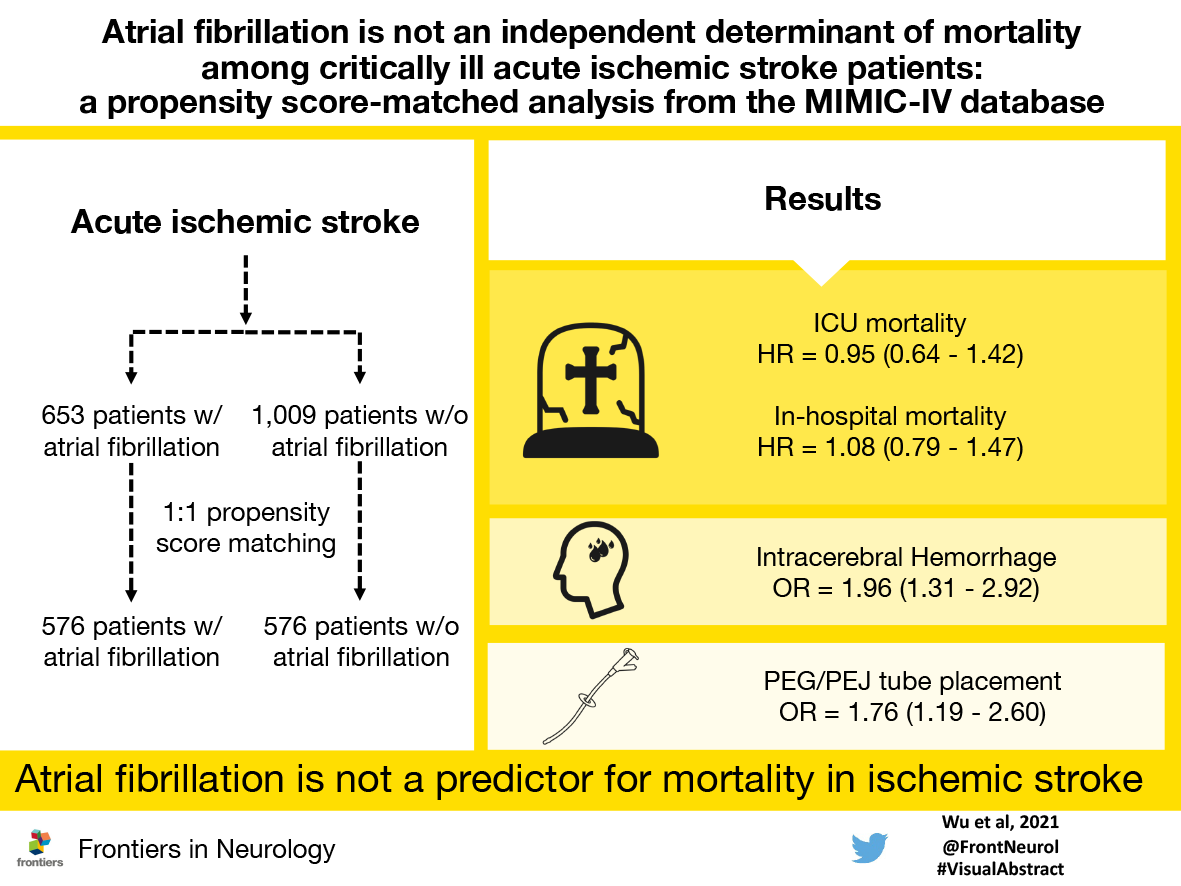

Supplement: Supplementary file 1 [file Image_1.TIF]
