# Supplementary material for: Atrial Fibrillation Is Not an Independent Determinant of Mortality Among Critically Ill Acute Ischemic Stroke Patients: A Propensity Score-Matched Analysis From the MIMIC-IV Database
Source: Front Neurol. 2022 Jan 17;12:730244. doi: 10.3389/fneur.2021.730244 (PMC8801535; doi:10.3389/fneur.2021.730244)
Supplement: Supplementary file 2 [file Table_1.docx]

Table S1 - ICD-9-CM and definition of CHA2DS2-VASc score

| Item | ICD 9 | ICD 10 | score |
| --- | --- | --- | --- |
| Congestive heart failure | 428, 398.91, 402.01, 402.11, 402.91, 404.01, 404.03, 404.11, 404.13, 404.91, 404.93, 425.4, 425.9 | I43, I50, I09.9, I11.0, I13.0, I13.2, I25.5, I42.0, I42.5, I42.6, I42.7, I42.8, I42.9, P29.0 | 1 |
| Hypertension | 401.1, 401.9, 402.10, 402.90, 404.10, 404.90, 405.11, 405.19, 405.91, 405.99 | I10, I11, I12, I13, I15, N26.2 | 1 |
| Age | NA | NA | 2, Age ≥ 75;  1, 75> Age ≥ 65;  0, Age < 65. |
| Diabetes mellitus | 250.0, 250.1, 250.2, 250.3,  250.8, 250.9, 250.4, 250.5, 250.6, 250.7 | E10.0, E10.l, E10.6, E10.8, E10.9, E11.0, E11.1, E11.6, E11.8, E11.9, E12.0, E12.1, E12.6, E12.8, E12.9, E13.0, E13.1, E13.6, E13.8, E13.9, E14.0, E14.1, E14.6, E14.8,  E14.9, E10.2, E10.3, E10.4, E10.5, E10.7, E11.2, E11.3, E114, E11.5, E11.7, E12.2, E12.3, E12.4, E12.5, E12.7, E13.2, E13.3, E13.4, E13.5, E13.7, E14.2, E14.3, E14.4, E145, E14.7 | 1 |
| Stroke, transient ischemic attack | 430, 431, 432.0, 432.1, 432.9, 433, 434, 436, 437.0, 437.1, | I60, I61, I62.0, I62.9, I63, I65, I66 | 2 |
| Vascular disease | MI: 410  PVD: 440.0, 440.9, 441.2, 441.4, 441.7, 441.9, 443.1, 443.9, 447.1, 557.1, 557.9, V43.4 | MI: I21, I22  PVD: E08. 51, E08.52, E09.51, E09.52, E10.51, E10.52, E11.51, E13.51, E13.52, I67.0, I70, I71, I73.1, I73.8, I73.9, I77.1, I77.71, I77.72, I77.73, I77.74, I77.79, I79, K55.1, K558, K559, Z95.82, Z95.9 | 1 |
| Gender | NA | NA | 2, Female; 1, Male. |
