# Supplementary material for: Atrial Fibrillation Is Not an Independent Determinant of Mortality Among Critically Ill Acute Ischemic Stroke Patients: A Propensity Score-Matched Analysis From the MIMIC-IV Database
Source: Front Neurol. 2022 Jan 17;12:730244. doi: 10.3389/fneur.2021.730244 (PMC8801535; doi:10.3389/fneur.2021.730244)
Supplement: Supplementary file 3 [file Table_2.docx]

Table S2 - ICD-9-CM and definition of HAS-BLED score

| Item | ICD 9 | ICD 10 | score |
| --- | --- | --- | --- |
| Hypertension | 401.1, 401.9, 402.10, 402.90, 404.10, 404.90, 405.11, 405.19, 405.91, 405.99 | I10, I11, I12, I13, I15, N26.2 | 1 |
| Renal disease | 582, 585, 586, V56,  588.0, V42.0, V45.1, 403.01,  403.11, 403.91, 404.02, 404.03, 404.12, 404.13, 404.92, 404.93, 583.0, 583.1, 583.2, 583.3, 583.4, 583.5, 583.6, 583.7 | N18, N19, I12.0, I13.1,  N03.2, N03.3, N03.4, N03.5, N03.6, N03.7, N05.2, N05.3, N05.4, N05.5, N05.6, N05.7,  N25.0, Z49.0, Z49.1, Z49.2, Z94.0, Z99.2 | 1 |
| Liver disease | 570, 571, 070.6, 070.9,  573.3, 573.4, 573.8, 573.9,  V42.7, 070.22, 070.23, 070.32, 070.33, 070.44, 070.54, 456.0, 456.1, 456.2, 572.2, 572.3, 572.4, 572.8 | B18, K73, K74, K70.0,  K70.1, K70.2, K70.3, K70.9, K71.3, K71.4, K71.5, K71.7, K76.0, K76/2, K76/3, K76.4,  K76.8, K76.9, Z94.4, I85.0, I85.9, I86.4, I98.2,  K70.4, K71.1, K72.1, K72.9, K76.5, K76.6, K76.7 | 1 |
| Stroke, transient ischemic attack | 430, 431, 432.0, 432.1, 432.9, 433, 434, 436, 437.0, 437.1, | I60, I61, I62.0, I62.9, I63, I65, I66 | 1 |
| Bleeding history | ICH:  430, 431, 432.0, 432.1, 432.9  Extra-cranial:  531.0, 531.2, 531.4, 531.6, 532.0, 532.2, 532.4, 532.6, 533.0, 533.2, 533.4, 533.6, 534.0, 534.2, 534.4, 534.6, 578.0, 578.1, 578.9, 569.3, 287.8, 287.9, 596.7, 784.8, 599.7, 627.1, 459.0, 719.1, 786.3 | ICH:  I60, I61, I62.0, I62.9  Extra-cranial:  K92.0, K92.1, I85.0, I98.20, I98.3, K22.10, K22.12, K22.14, K22.16, K25.0, K25.2, K25.4, K25.6, K26.0, K26.2, K26.4, K26.6, K27.0, K27.2, K27.4, K27.6, K28.0, K28.2, K28.4, K28.6, K29.0, K63.80, K31.80, K55.20, K62.5, K92.2, N02.0, N02.1, N02.2, N02.3, N02.4, N02.5, N02.6, N02.7, N02.8, N02.9, K66.1, N93.8, N93.9, N95.0, R04.1, R04.2, R04.8, R04.9, R31.0, R31.1, R31.8, R58, D68.3, H35.6, H43.1, H45.0, M25.0 | 1 |
| INR > 4 | NA | NA | 1 |
| Age | NA | NA | 1, age ≥ 65;  0, age < 65. |
| Medication usage predisposing to bleeding | NA | NA | 1 |
| Alcohol use | 291.1, 291.2, 291.5, 291.8, 291.9, 303.93, 305.03, V11.3 | F10, E52, G62.1, I42.6, K29.2, K70.0, K70.3, K70.9, T51, Z71.4, Z65.8 | 1 |
