# Supplementary material for: Atrial Fibrillation Is Not an Independent Determinant of Mortality Among Critically Ill Acute Ischemic Stroke Patients: A Propensity Score-Matched Analysis From the MIMIC-IV Database
Source: Front Neurol. 2022 Jan 17;12:730244. doi: 10.3389/fneur.2021.730244 (PMC8801535; doi:10.3389/fneur.2021.730244)
Supplement: Supplementary file 4 [file Table_3.docx]

Table S3 - ICD-9-CM and definition of Charlson comorbidity Index

| Condition | ICD 9 | ICD 10 | Weight |
| --- | --- | --- | --- |
| Age score | NA | NA | 4, Age ≥ 70; 3, 70 > Age ≥ 61;  2, 60 > Age ≥ 51; 1, 50 > Age ≥ 41;  0, Age < 40. |
| Myocardial infarct | 410, 412 | I21, I22, I25.2 | 1 |
| Congestive heart failure | 428, 398.91, 402.01, 402.11, 402.91, 404.01, 404.03, 404.11, 404.13, 404.91, 404.93, 425.4, 425.9 | I43, I50, I09.9, I11.0, I13.0, I13.2, I25.5, I42.0, I42.5, I42.6, I42.7, I42.8, I42.9, P29.0 | 1 |
| Peripheral vascular disease | 440, 441, 093.0, 437.3, 447.1, 557.1, 557.9, V43.4, 443.1, 443.9 | I70, I71, I73.1, I73.8, I73.9, I77.1, I79.0, I79.2,  K55.1, K55.8, K55.9, Z95.8, Z95.9 | 1 |
| Cerebrovascular disease | 430, 431, 432, 433, 434, 435, 436, 437, 438, 362.34 | G45, G46, I60~I69, H34.0 | 1 |
| Dementia | 290, 294.1, 331.2 | F00, F01, F02, F03,  G30, F051, G311 | 1 |
| Chronic pulmonary disease | 490, 491, 492, 493, 494, 495, 496, 500, 501, 502, 503, 504, 505, 4168, 4169, 506.4, 508.1, 508.8 | J40~J47, J60~J67, I27.8, I27.9, J68.4, J70.1,  J703 | 1 |
| Rheumatic disease | 725, 446.5, 710.0, 710.1,  710.2, 710.3, 710.4, 714.0,  714.1, 714.2, 714.8 | M05, M06, M32, M33,  M34, M31.5, M35.1, M35.3, M36.0 | 1 |
| Peptic ulcer disease | 531, 532, 533, 534 | K25, K26, K27, K28 | 1 |
| Mild liver disease | 570, 571, 070.6, 070.9,  573.3, 573.4, 573.8, 573.9,  V42.7, 070.22, 070.23, 070.32, 070.33, 070.44, 070.54 | B18, K73, K74, K70.0,  K70.1, K70.2, K70.3, K70.9, K71.3, K71.4, K71.5, K71.7, K76.0, K76/2, K76/3, K76.4,  K76.8, K76.9, Z94.4 | 1 |
| Diabetes without chronic complication | 250.0, 250.1, 250.2, 250.3,  250.8, 250.9 | E10.0, E10.l, E10.6, E10.8, E10.9, E11.0, E11.1, E11.6, E11.8, E11.9, E12.0, E12.1, E12.6, E12.8, E12.9, E13.0, E13.1, E13.6, E13.8, E13.9, E14.0, E14.1, E14.6, E14.8,  E14.9 | 1 |
| Diabetes with chronic complication | 250.4, 250.5, 250.6, 250.7 | E10.2, E10.3, E10.4, E10.5, E10.7, E11.2, E11.3, E114, E11.5, E11.7, E12.2, E12.3,  E12.4, E12.5, E12.7, E13.2, E13.3, E13.4, E13.5, E13.7, E14.2, E14.3, E14.4, E145,  E14.7 | 2 |
| Paraplegia | 342, 343, 3341, 344.0,  344.1, 344.2, 344.3, 344.4,  344.5, 344.6, 344.9 | G81, G82, G04.1, G11.4,  G80.1, G80.2, G83.0, G83.1, G83.2, G83.3, G83.4, G83.9 | 2 |
| Renal disease | 582, 585, 586, V56,  588.0, V42.0, V45.1, 403.01,  403.11, 403.91, 404.02, 404.03, 404.12, 404.13, 404.92, 404.93, 583.0, 583.1, 583.2, 583.3, 583.4, 583.5, 583.6, 583.7 | N18, N19, I12.0, I13.1,  N03.2, N03.3, N03.4, N03.5, N03.6, N03.7, N05.2, N05.3, N05.4, N05.5, N05.6, N05.7,  N25.0, Z49.0, Z49.1, Z49.2, Z94.0, Z99.2 | 2 |
| Malignant cancer | 140~172, 1740~1958, 200~208, 238.6 | C43,C88, C00~C26, C30~C34, C37~C41, C45~C58, C60~C76, C81~C85, C90~C97 | 2 |
| Severe liver disease | 456.0, 456.1, 456.2, 572.2, 572.3, 572.4, 572.8 | I85.0, I85.9, I86.4, I98.2,  K70.4, K71.1, K72.1, K72.9, K76.5, K76.6, K76.7 | 3 |
| Metastatic solid tumor | 196, 197, 198, 199 | C77, C78, C79, C80 | 6 |
| HIV/AIDS | 042, 043, 044 | B20, B21, B22, B24 | 6 |
