# Supplementary material for: Atrial Fibrillation Is Not an Independent Determinant of Mortality Among Critically Ill Acute Ischemic Stroke Patients: A Propensity Score-Matched Analysis From the MIMIC-IV Database
Source: Front Neurol. 2022 Jan 17;12:730244. doi: 10.3389/fneur.2021.730244 (PMC8801535; doi:10.3389/fneur.2021.730244)
Supplement: Supplementary file 5 [file Table_4.docx]

| Table S4. Sensitivity analysis regarding the association between outcomes and atrial fibrillation | | | | | | |
| --- | --- | --- | --- | --- | --- | --- |
|  | With Atrial Fibrillation versus Without atrial Fibrillation *(Reference)* | | | | | |
|  | Before PSM - Univariate | | After PSM - Univariate | | After PSM - Multivariate | |
| Outcomes | Crude HR  (95%CI) | *P* value | Crude HR  (95%CI) | *P* value | Adjusted HR  (95%CI) # | *P* value |
| ICU Mortality | 1.36 (0.97 - 1.91) | 0.076 | 0.98 (0.66 - 1.45) | 0.915 | 1.00 (0.66 - 1.52) | 0.986 |
| In-hospital Mortality | 1.57 (1.20 - 2.05) | 0.001 | 1.03 (0.76 - 1.38) | 0.868 | 1.16 (0.85 - 1.60) | 0.350 |
| Outcomes | Crude OR  95%CI | *P* value | Crude OR  95%CI | *P* value | Adjusted OR  95%CI | *P* value |
| Intracerebral Hemorrhage | 2.16 (1.60 - 2.93) | <0.001 | 1.78 (1.25 - 2.54) | 0.002 | 1.77 (1.19 - 2.63) | 0.005 |
| PEG/PEJ tube placement | 2.03 (1.51 - 2.72) | <0.001 | 1.85 (1.30 - 2.62) | 0.001 | 1.72 (1.16 - 2.56) | 0.007 |

Propensity score matching by age, sex, Charlson comorbidity Index, acute physiology score III, the CHA2DS2-VASc score, and HAS-BLED score.

HR: hazard ratio; OR: odds ratio; PEG: percutaneous endoscopic gastrostomy; PEJ: percutaneous endoscopic jejunostomy.

#All results of HR/OR were adjusted by race, hypertension, congestive heart failure, liver disease, diabetes, anti-platelet agents, anti-coagulation agents (warfarin and novel oral anticoagulant), intravenous tissue plasminogen activator or endovascular mechanical thrombectomy.
